# Inhibition of Rac1-dependent forgetting alleviates memory deficits in animal models of Alzheimer’s disease

**DOI:** 10.1007/s13238-019-0641-0

**Published:** 2019-07-18

**Authors:** Wenjuan Wu, Shuwen Du, Wei Shi, Yunlong Liu, Ying Hu, Zuolei Xie, Xinsheng Yao, Zhenyu Liu, Weiwei Ma, Lin Xu, Chao Ma, Yi Zhong

**Affiliations:** 1 Tsinghua-Peking Center for Life Science, IDG/McGovern Institutes for Brain Research, MOE Key Laboratory of Protein Science, School of Life Sciences, Tsinghua University, Beijing, 100084 China; 2 Life Science Division, Graduate school at Shenzhen, Tsinghua University, Shenzhen, 518055 China; 3 JoeKai Biotech. LLC, LianQiang International Building, Yongfeng Base, Beijing, 100084 China; 4 Institute of Traditional Chinese Medicine and Natural Products, College of Pharmacy, Jinan University, Guangzhou, 510632 China; 5 Key Lab of Animal Models and Human Disease Mechanisms, Kunming Institute of Zoology, Chinese Academy of Sciences, Kunming, 650223 China; 6 Institute of Basic Medical Sciences, Chinese Academy of Medical Sciences, Department of Human Anatomy, Histology and Embryology, Neuroscience Center, Joint Laboratory of Anesthesia and Pain, School of Basic Medicine, Peking Union Medical College, Beijing, 100005 China

**Keywords:** Alzheimer’s disease, Rac1, forgetting, memory loss, hippocampus

## Abstract

Accelerated forgetting has been identified as a feature of Alzheimer’s disease (AD), but the therapeutic efficacy of the manipulation of biological mechanisms of forgetting has not been assessed in AD animal models. Ras-related C3 botulinum toxin substrate 1 (Rac1), a small GTPase, has been shown to regulate active forgetting in *Drosophila* and mice. Here, we showed that Rac1 activity is aberrantly elevated in the hippocampal tissues of AD patients and AD animal models. Moreover, amyloid-beta 42 could induce Rac1 activation in cultured cells. The elevation of Rac1 activity not only accelerated 6-hour spatial memory decay in 3-month-old APP/PS1 mice, but also significantly contributed to severe memory loss in aged APP/PS1 mice. A similar age-dependent Rac1 activity-based memory loss was also observed in an AD fly model. Moreover, inhibition of Rac1 activity could ameliorate cognitive defects and synaptic plasticity in AD animal models. Finally, two novel compounds, identified through behavioral screening of a randomly selected pool of brain permeable small molecules for their positive effect in rescuing memory loss in both fly and mouse models, were found to be capable of inhibiting Rac1 activity. Thus, multiple lines of evidence corroborate in supporting the idea that inhibition of Rac1 activity is effective for treating AD-related memory loss.

**Electronic supplementary material:**

The online version of this article (10.1007/s13238-019-0641-0) contains supplementary material, which is available to authorized users.

## Introduction

The pathological hallmarks of Alzheimer’s disease (AD) are excessive accumulation of senile plaques (SPs) and neurofibrillary tangles (NFTs) in the brain, leading to synaptic dysfunction and cognitive deficits (Hardy and Selkoe, 2002; Ballatore et al., 2007). Although memory loss, an initial symptomatic feature, has been reported extensively (Reitz et al., 2011), the cognitive nature of this memory loss is not well understood. The rapid forgetting theory was proposed in neuropsychological studies (Hart et al., 1988; Salmon et al., 1989). Recently, in autosomal dominant (familial) AD, accelerated forgetting was identified as a presymptomatic feature (Weston et al., 2018). However, the use of pharmacological interventions to suppress forgetting, as a potential treatment for AD, has not yet been tested because of the lack of an understanding of the biological mechanisms underlying forgetting. Recent studies on Rac1, a member of the Rho family of small GTPases, may shed light on this matter.

Rac1 acts as a molecular switch that regulates synaptic structural and functional plasticity by dynamically controlling the reorganization of the actin cytoskeleton (Etienne-Manneville and Hall, 2002). Rac1 is identified as a key molecular component in mediating active forgetting (Shuai et al., 2010). Learning not only leads to memory formation, but also evokes Rac1-dependent active forgetting to regulate memory decay (Shuai et al., 2010; Davis and Zhong, 2017). This mechanism is conserved in several organisms; from *Drosophila* (Shuai et al., 2010; Cervantes-Sandoval et al., 2016) to mice (Hayashi-takagi et al., 2015; Liu et al., 2016; Liu et al., 2018). The stability of long-term potentiation (LTP) can also be bidirectionally regulated by Rac1 activity (Liu et al., 2016).

Amyloid-beta 42 (Aβ42), an established neurotoxic peptide involved in AD, can activate Rac1 in cultured hippocampal neurons (Mendoza-Naranjo et al., 2007) and in SN4717 cells (Manterola et al., 2013) through different pathways. However, the effect of the prevention of Rac1-dependent forgetting on cognitive impairment in AD has not been studied. In this study, we inhibited Rac1 activity in AD animal models using genetic and pharmacological approaches to assess its therapeutic effect. Our data indicate that the inhibition of Rac1 activity is a valuable strategy for ameliorating memory loss in AD models.

## Results

### Enhanced Rac1 activity in AD patients and animal models

**Figure 1:**
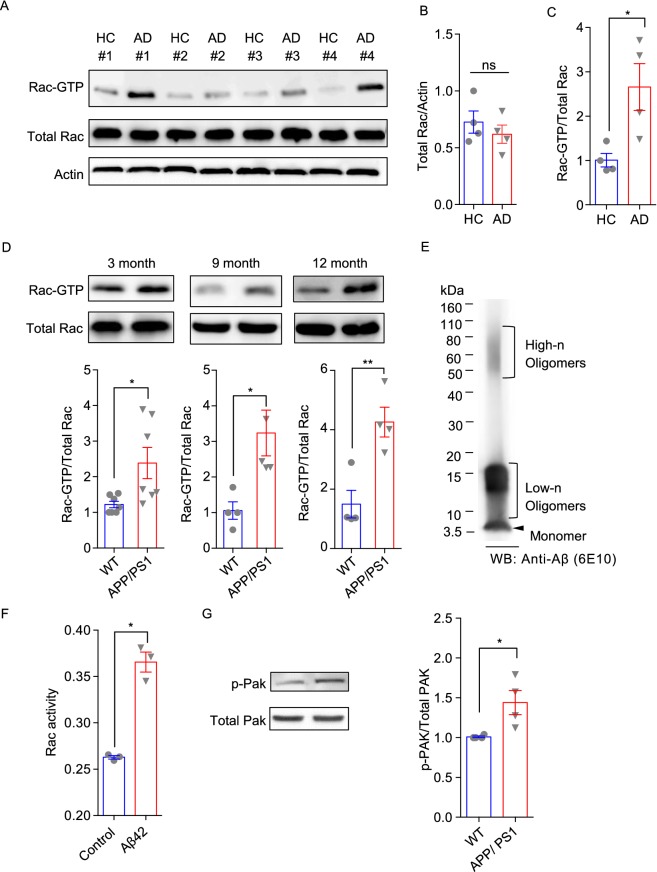
**Elevation of Rac1 activity in both AD patients and AD animal models**. (A) Western blot of Rac1-GTP, Total Rac1 and actin in the hippocampal tissues of AD patients and age-matched, non-demented healthy controls (HC). Detailed information on the human samples is provided in Table 1. (B) The amount of Rac1 showed no significant difference between AD patients and HC (ns, *P* > 0.05, *t*-test, two-tailed, *n* = 4 for each group; same sample numbers as (A)). (C) Rac1-GTP/Total Rac1 ratio in AD patients was significantly higher than that in HC (*P < 0.05, *t*-test, two-tailed, *n* = 4 for each group; same sample numbers as (A)). (D) Hippocampal tissue was collected from mice of different ages for Western blot. A representative Western blot is shown in the upper panel. Rac1-GTP/Total Rac1 ratio (Rac1 activity) was significantly higher in APP*swe*/PS1*dE9* (APP/PS1) mice than that in wild-type (WT) at 3, 9 and 12 months old (*P < 0.05, **P < 0.01; ns, *P* > 0.05, *t*-test, two-tailed; *n* = 7 for 3-month-old mice, *n* = 4 for 9- and 12-month-old mice). (E) Western blot to assess the structure and size of Aβ42 prepared for the *in vitro* Rac1 activation assay. Anti-Aβ42 monoclonal antibody 6E10 was used. (F) Aβ42-activated Rac1 in HEK293 cells detected by the Rac1 GLISA Kit (*P < 0.05, *t*-test, two-tailed; *n* = 3 for each group). (G) A representative Western blot of phosphorylated PAK and total PAK is shown (left panel). The activity of PAK increased significantly in the hippocampal tissue of 9-month-old APP/PS1 mice (right panel, *P < 0.05, *t*-test, two-tailed; *n* = 4 for each group). Data information: All values are expressed as mean ± SEM

We first assayed Rac1 activity in hippocampal tissues from AD patients and healthy age-matched, non-demented controls (Detailed information is provided in Table 1). The levels of the guanosine triphosphate (GTP)-bound active form of Rac1 were measured using the p21-binding domain (PBD) pull-down assay. The GTP-bound active form of Rac1 was pulled down through the interactive p21-activated kinase (PAK) fragment using PAK-PBD beads and measured using a Western blot assay (Fig. 1A), as described in previous studies (Shuai et al., 2010; Liu et al., 2016) from our laboratory and studies conducted by other research groups (Sander et al., 1998; Barnes et al., 2008). The total amount of Rac1 and actin were also evaluated using the Western blot analyses (See MATERIALS AND METHODS). The total Rac1 level relative to actin level in humans was not significantly different between the AD patients and healthy controls (Fig. 1B). Since there were no significant differences in total Rac1 levels between the experimental and control groups (See representative Western blot in all relevant figures), the ratio of Rac1-GTP to total Rac1 was taken as a measure of Rac1 activity. We found that Rac1 activity increased 2.5-fold in AD patients compared to that in healthy controls (Fig. 1C). Consistent with this observation, Rac1 activity was also elevated in the hippocampus of APP/PS1 mice at different age (Fig. 1D). Furthermore, the activity of PAK, a downstream molecule of Rac1, increased significantly in the hippocampal tissue of 9-month-old APP/PS1 mice (Fig. 1G). Similarly, Rac1 activity was significantly increased in the whole brain of transgenic flies 5 and 10 days after eclosion (DAE) with pan-neuronal expression of a secreted form of Aβ42. This increase was more pronounced in older flies (Fig. S1F).

**Table 1:** General information of Alzheimer’s disease patients and healthy controls (HC).

ID	Gender	Age	Medical history	Sampling delay (h)
AD group				
PTB048 (AD#1)	F	82	Emphysema, mild AD	4
PTB050 (AD#2)	F	80	Cerebral thrombosis, pulmonary embolism, AD	4.5
PTB051 (AD#3)	M	89	Chronic bronchitis, arrhythmia, moderate AD (Phase III)	5
PTB078 (AD#4)	F	86	Organ failure, Mild AD	6.33
Control group				
PTB044 (HC#1)	F	85	Primary liver cancer, hypertension	7.5
PTB055 (HC#2)	F	84	Coronary disease	5
PTB080 (HC#3)	F	86	Diabetes, coronary disease, cerebral thrombosis	4.5
PTB087 (HC#4)	M	78	Diabetes, cirrhosis	8

Rac1 activity is elevated not only in AD patients, but also in both Aβ cascade-based mouse and fly models. For this reason, we investigated the possible role of Aβ42 in enhancing Rac1 activity. The size and structure of Aβ42 was assessed using the 6E10 antibody (Fig. 1E). In cultured HEK-293 cells, oligomers of Aβ42 were capable of stimulating Rac1 activity when they were directly applied on the culture medium (Fig. 1F), suggesting that an age-dependent increase in Rac1 activity may be linked to age-dependent Aβ42 accumulation. This observation motivated us to investigate the role of Rac1 in age-dependent memory loss in both mouse and fly animal models.

### Rac1-dependent forgetting contributes to age-dependent memory loss

**Figure 2:**
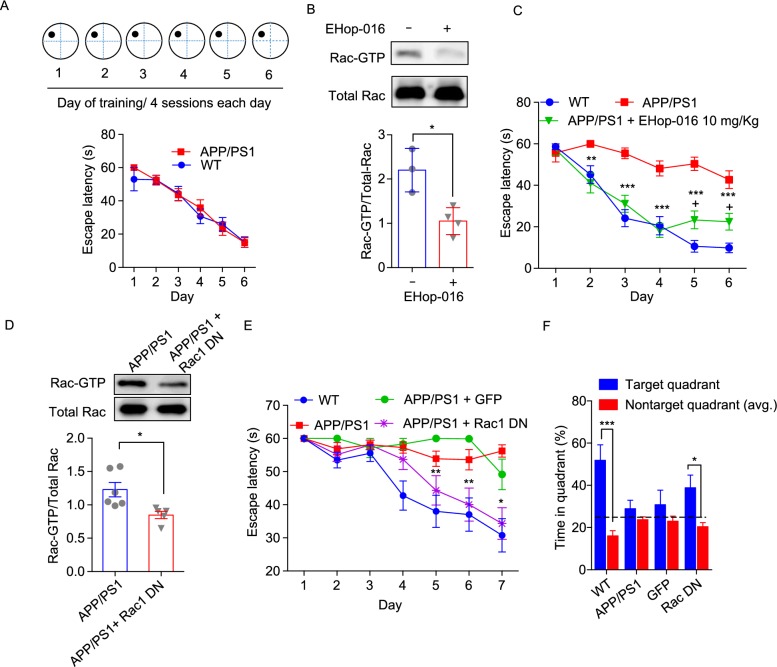
**The ameliorating effect of Rac1 activity inhibition on the learning defects associated with APP/PS1**. (A) No learning deficits were observed in the young (3-month-old) APP/PS1 mice in comparison with age-matched WT mice in the Morris Water Maze (MWM) assay. The training paradigm (upper panel); escape latency curve during the MWM task (lower panel) (*P* > 0.05, two-way ANOVA followed by Sidak’s multiple comparison test; *n* = 7 per group). (B) Western blot of EHop-016 showed inhibition of Rac1 activity in the hippocampal tissues of 9-month-old AD mice. Comparison of Rac1 activity in hippocampal tissues after administration of EHop-016 (10 mg/kg) for 10 days (**P < 0.01, ***P < 0.0001, *t*-test, two-tailed; *n* = 3 for the EHop-016(-) control group, *n* = 4 for the EHop-016(+) treatment group). (C) Administration of Rac1 activity inhibitor (EHop-016) mitigated the learning deficit in 9-month-old APP/PS1 mice. Both WT control group (blue line) and APP/PS1 control group (red line) received the same volume of vehicle throughout the assay. Escape latency curve during the MWM (+*P* < 0.05, two-way ANOVA followed by Tukey’s multiple comparison test, treatment effect, APP/PS1 control versus APP/PS1 + EHop-016; **P < 0.01, ***P < 0.0001, two-way ANOVA followed by Tukey’s multiple comparison test; *n* = 7 per group, genotype effect, WT control versus APP/PS1 + EHop-016). (D) Rac1 activity was significantly suppressed in hippocampal tissues after injection of rAAV-CaMKIIα-Rac1 DN or PBS (*P < 0.05, *t*-test, two-tailed; *n* = 5 per group). (E) Escape latency curve during the MWM task. Genetic manipulation of Rac1 activity in the hippocampus by the specific expression of dominant negative Rac1 (Rac1 DN) in 12-month-old APP/PS1 mice with rescued learning defect. Both the WT control group (blue line) and APP/PS1 control group (red line) underwent sham surgeries (*P < 0.05, **P < 0.01, two-way ANOVA followed by Tukey’s multiple comparison test; *n* = 7 per group). (F) Percentage of time spent in the target quadrant during the probe test. GFP: APP/PS1 + GFP; Rac1 DN: APP/PS1 + Rac1 DN (*P < 0.05, ***P < 0.0001, *t*-test, two-tailed, same mice as in (E)). Data information: All values are expressed as mean ± SEM

It is well documented that during the Morris water maze (MWM) task, the spatial learning curve is defective only in old APP/PS1 mice (mice over seven months old) but not in younger APP/PS1 littermates (3-month-old mice) (Trinchese et al., 2004; Zhang et al., 2012). As seen in Figure 2A–C, the learning curve was completely normal in 3-month-old mice but was defective in 9-month-old APP/PS1 mice. The administration of EHop-016 (10 mg/kg), a Rac inhibitor (Montalvo-Ortiz et al., 2012), significantly inhibited Rac1 activity without changing total Rac1 expression in the hippocampal tissues of 9-month-old AD mice (Fig. 2B). Accordingly, EHop-016 (10 mg/kg)-treated AD mice took less time to locate the hidden platform compared to their vehicle-treated AD counterparts (Fig. 2C). Importantly, cognitive performance in EHop-016-treated AD mice did not significantly differ from that of WT mice (*P* > 0.05), except on days 5 and 6 (*P* < 0.05).

To examine the specificity of the observed EHop-016 rescuing effect, the effect of recombinant adeno-associated virus (rAAV)-mediated genetic manipulation of Rac1 activity in the hippocampus was investigated. The injection location is shown in Fig. S2A. Rac1 activity was significantly suppressed in AD mice injected with rAAV-CaMKIIα-Rac-dominant negative (DN) (Fig. 2D). Correlated with this reduction, the learning curve defect was also rescued (Fig. 2E). The impact of Rac1 activity manipulation on memory retention was examined using the probe test. The Rac1 DN group spent a greater percentage of time in the target quadrant compared to that in the nontarget quadrant (Fig. 2F).

The rescue effect was interesting because the observed cognition impairment phenotype is present as a defect in the learning curve during the MWM task, whereas manipulation of the Rac1 pathway in the hippocampus supposedly does not influence learning or memory formation, but instead specifically regulates memory retention (Shuai et al., 2010; Liu et al., 2016). This led to the hypothesis that the observed learning defect in original paradigms resulted from accelerated forgetting.

**Figure 3:**
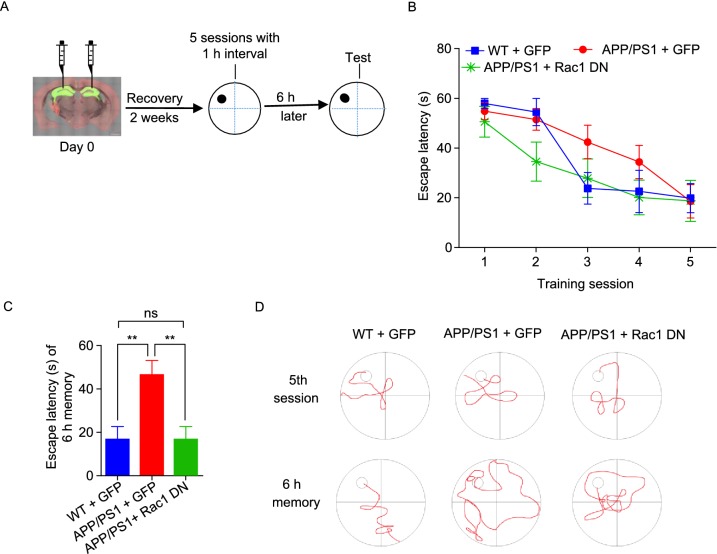
**Accelerated spatial memory loss in young (3-month-old) APP/PS1 mice could be halted by expression of Rac1 DN in the hippocampus**. (A) Schematic depiction of the time course for the injection of rAAV-CaMKIIα driven Rac1 DN and the modified MWM task. (B) All groups of mice exhibited the same strength of spatial memory after five training sessions within one day. WT (blue line) and APP/PS1 (red line) as control groups underwent sham surgeries (*P* > 0.05, two-way ANOVA followed by Tukey’s multiple comparison test; *n* = 7 per group). (C) Escape latency for 6-hour memory test. The expression of Rac1 DN in the hippocampus halted the accelerated memory loss in APP/PS1 (**P < 0.05; ns, *P* > 0.05, one-way ANOVA following post-hoc analysis). (D) A representative image showing the movements of mice in each group at the fifth session and at the 6-hour memory test. Data information: All values are expressed as mean ± SEM

To test this hypothesis, we examined younger (3-month-old) mice with intact learning ability (Fig. 2A) using a modified MWM paradigm (Fig. 3A). As expected, differences in the learning curve, obtained from five consecutive training sessions massed together, were statistically insignificant for all three groups (Fig. 3B). Spatial memory was retained at 2 and 4 h after multiple trainings in a single day (Fig. S2B), but had decayed significantly after 6 h in the APP/PS1 control group (escape latency, Fig. 3C; representative swimming trace, Fig. 3D) compared to the well-retained spatial memory in the WT control group. This accelerated memory decay was rescued in APP/PS1 mice following the inhibition of Rac1 activity through the expression of Rac1-DN.

**Figure 4:**
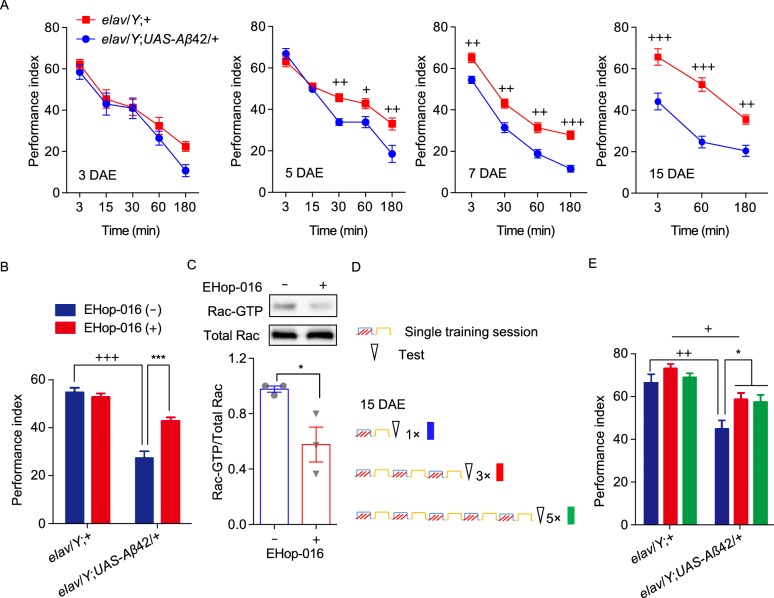
**Rac1-dependent forgetting contributes to memory loss in an age-dependent manner in the Tg-AD fly model**. (A) Memory curves of AD flies at different ages (days after eclosion, DAE), (+*P* < 0.05, ++*P* < 0.01, +++*P* < 0.0001, two-way ANOVA followed by Tukey’s multiple comparison test, 3 DAE: *n* = 6, 5 DAE: *n* = 12, 7 DAE: *n* = 6, 15 DAE: *n* = 10). (B) Administration of EHop-016 rescued the learning defect (***P < 0.0001, treatment effect; +++*P* < 0.0001, genotype effect. One-way ANOVA followed by post-hoc analysis; *n* = 6 for *elav*/*Y*;+ fed with EHop-016(-); *n* = 6 for *elav*/*Y*;+ fed with EHop-016(+); n = 8 for *elav*/*Y*; *UAS-Aβ42*/+ fed with EHop-016(-); *n* = 12 for *elav*/*Y; UAS-Aβ42*/+ fed with EHop-016(+)). (C) Western blot of EHop-016 showed inhibition of Rac1 activity in whole brain lysis (*P < 0.05, *t*-test, two-tailed; *n* = 3 per group). (D) Schematic paradigm of multiple training of AD flies. (E) Multiple training partially rescued the learning defects in 15 DAE AD flies. Performance after multiple training (*P < 0.05, treatment effect; +*P* < 0.05, ++*P* < 0.01, genotype effect; One-way ANOVA followed by post-hoc analysis; *n* = 10 for each group of *elav*/*Y*;+ and *elav*/*Y*; *UAS-Aβ42*/+ with 1× and 3× training; *n* = 12 for each group of *elav*/*Y*;+ and *elav*/*Y*; *UAS-Aβ42*/+ with 5× training). Data information: All values are expressed as mean ± SEM

Age-dependent accelerated forgetting was also observed in the AD fly. Three days after eclosion in the AD fly, learning was normal, and memory was comparable to that in the controls for up to 60 min. After 180 min, the memory scores of AD flies were lower than those in the controls, but the difference was not statistically significant. Five days after eclosion, AD flies still demonstrated normal learning, but the memory after 30 min, 60 min, and 180 min was significantly lost. Seven and fifteen days after eclosion, the flies showed learning defects (Fig. 4A). Administration of 100 μmol/L of EHop-016 significantly rescued the learning defects (Fig. 4B). Accordingly, EHop-016 administration in AD flies reduced Rac1 activity without changing the total Rac1 expression (Fig. 4C). Consistent with the drug’s effects, repeated training rescued the learning defect (Fig. 4E) as it can also inhibit Rac1 activity (Shuai et al., 2010).

### Rescue of long-term potentiation defects through inhibition of Rac1 activity

**Figure 5:**
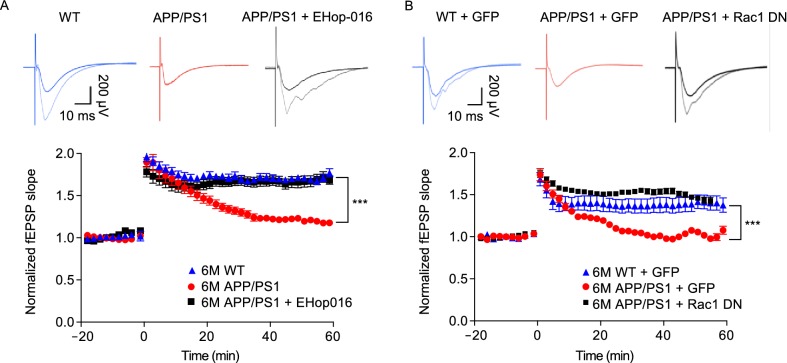
**Deficit of synaptic plasticity in APP/PS1 is ameliorated by inhibition of Rac1**. (A) Representative example of evoked responses immediately before (solid line) and 30 min after (dashed line) induction of LTP (upper panel). Deficit of LTP maintained in 6-month-old APP/PS1 mice was attenuated by EHop-016 (lower panel, ***P < 0.0001, two-way ANOVA followed by Tukey’s multiple comparison test, *n* = 14 for WT, *n* = 13 for APP/PS1, *n* = 13 for APP/PS1 + EHop-016). (B) Representative examples of evoked responses immediately before (solid line) and 30 min after (dashed line) induction of LTP (upper panel). Deficit of LTP maintained in 6-month-old APP/PS1-GFP mice was rescued by expression of Rac1 DN in the hippocampus (lower panel, ***P < 0.0001, two-way ANOVA followed by Tukey’s multiple comparison test, *n* = 14 for WT-GTF, *n* = 13 for APP/PS1 + GFP, *n* = 12 for APP/PS1-Rac1 DN). Data information: All values are expressed as mean ± SEM

The data presented above support the significant role of active forgetting in AD-associated memory loss at the molecular and behavioral levels. To investigate the association between active forgetting and memory loss at the cellular level, we recorded long-term potentiation (LTP), which is believed to be the cellular analog of learning and memory (Bliss and Collingridge, 1993). It is generally accepted that LTP is defective in Aβ-based AD models (Nalbantoglu et al., 1997; Klyubin et al., 2005). In the present study, field potential recordings showed LTP maintenance defects in the brain slices of six-month-old AD mice. LTP induction through theta burst stimulation (TBS) appeared to be normal but decayed rapidly. The representative voltage traces are shown (Fig. 5A and Fig. 5B upper panels). The fast decay was rescued by adding EHop-016 (10 μmol/L) to the artificial cerebrospinal fluid (ACSF) at the start of the recording (Fig. 5A lower panel). The defect was also rescued in brain slices from AAV-injected mice following the expression of Rac1-DN (*P* < 0.05, Fig. 5B lower panel). This confirms that the inhibition of Rac1 activity rescues accelerated LTP decay in AD mice.

### Multiple compounds identified through behavioral screening inhibit Rac1 activity

**Figure 6:**
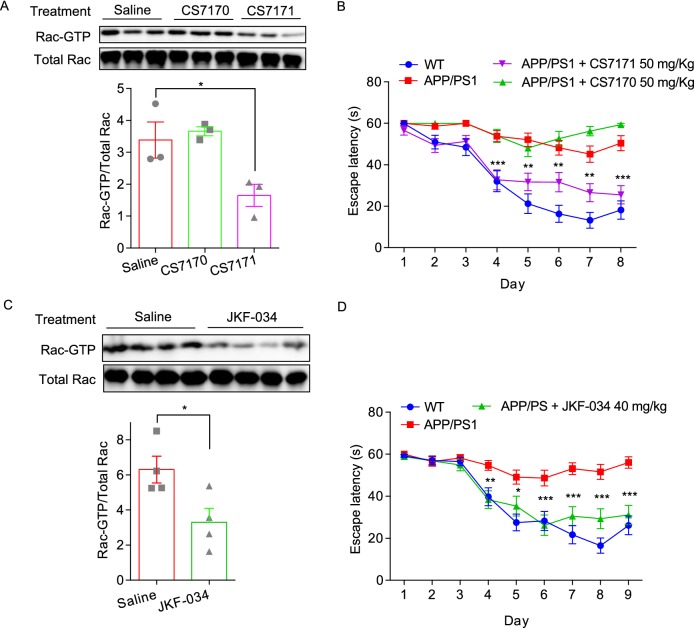
**Two compounds rescue learning deficits in APP/PS1 mice by inhibiting Rac1 activity in the hippocampus**. (A) Western blot of Rac1-GTP and total Rac1 in the hippocampus of APP/PS1 mice after oral administration of CS7171 and CS7170 (*P < 0.05, one-way ANOVA followed by Tukey’s multiple comparison test; *n* = 3 per group). (B) Learning deficit was rescued by CS7171, but not by CS7170. WT control group and APP/PS1 control group received vehicle during the assay (**P < 0.01, ***P < 0.001, two-way ANOVA followed by Tukey’s multiple comparison test; *n* = 7 per group). (C) Western blot of Rac1-GTP and total Rac1 in the hippocampus from APP/PS1 mice after oral feeding with JKF-034 (*P < 0.05, *t*-test, two-tailed; *n* = 4 per group). (D) Learning deficit was rescued by JKF-034. The WT control group and APP/PS1 control group received vehicle during the assay (*P < 0.05, **P < 0.01, ***P < 0.001, two-way ANOVA followed by Tukey’s multiple comparison test; *n* = 7 per group). Data information: All values are expressed as mean ± SEM

As reported previously (Wang et al., 2012), we performed behavioral screening of over 2,000 synthetic compounds that presumably target protein kinases. Each compound was fed to flies once per day for seven consecutive days. The flies underwent aversive olfactory conditioning (see the Materials and Methods 2.8) 10 DAE. The identified effective compounds were tested in APP/PS1 mice while they were performing the MWM task. Based on the aforementioned findings, we assessed the effects of these identified compounds, which were shown to be effective in rescuing memory loss in both APP/PS1 mouse and AD fly models, on Rac1 activity. Surprisingly, a large number of these compounds was capable of inhibiting Rac1 activity. Two examples of these compounds are presented in Fig. 6. Two compounds with highly similar structures, CS7171 and CS7170, provided evidence for an association between Rac1 activity and memory loss (Fig. S3A–C). CS7171, but not CS7170, inhibited hippocampal Rac1 activity and mitigated learning deficits in eight-month-old APP/PS1 mice (Fig. 6A and 6B). We also identified another effective compound, JKF-034 (Fig. 6C and 6D), with a different structure (Fig. S3C). Three important pharmacological assays were conducted. They were the *in vitro* blood-brain barrier (BBB) permeability assay, the pharmacokinetics (PK) assay, and the *in vitro* metabolism stability in liver microsome assay. As shown in Table 2, JKF-034 had a high permeability coefficient (P_app_) (A-B, 57.33 × 10^−6^ cm/s), while that of CS7171 was moderate (A-B, 7.91 × 10^−6^ cm/s). These results suggest that JKF-034 is more efficient at crossing the BBB. The two compounds were absorbed rapidly after oral administration, with the time to reach the maximum plasma concentration (T_max_) ranging from 0.25 to 5 h. The metabolic half-time (T_1/2_) of JKF-034 was about ten times higher than that of CS7171. The *invivo* bioavailabilities (F) of CS7171 and JKF-034 were 28.2 and 19.3, respectively. The relatively high F value indicates that CS7171 might have favorable drug-like properties (Table 3). The T_1/2_ of CS7171 and JKF-034 in liver microsomes were less than 30 min (Table 4). The low T_1/2_ values of CS7171 and JKF-034 suggest that these compounds are susceptible to metabolism in liver microsomes.

**Table 2:** Permeability coefficient of CS7171 and JKF-034 in the *in vitro* blood-brain barrier permeability assay.

Test article		P_app_ (10^−6^ cm/s)	
		Mean	RSD
Metoprolol	A-B	40.55	0.11
	B-A	28.60	0.05
Atenolol	A-B	<2.09	N/A
	B-A	<1.02	N/A
CS7171	A-B	7.91	0.09
	B-A	3.61	0.04
JKF-034	A-B	57.33	0.06
	B-A	32.79	0.01

**Table 3:** Pharmacokinetic parameters of CS7171 and JKF-034 in CD1 mice.

Parameters	Analytes (Estimated value, *n* = 3)	
	CS7171	JKF-034
T_max_ (h)	0.25	0.5
T_1/2_ (h)	0.943	9.19
AUC0-t (h*ng/mL)	4,101	1,262
AUC0-infinity (h*ng/mL)	4,160	1,692
F (%)	28.2	19.3

AUC: area under curve

**Table 4:** Metabolic stability of CS7171 and JKF-034 in liver microsomes.

Test article	Species	T_1/2_ (min)	Cl_int_ (mL/min/kg)
Ketanserin	Human	44.06	39.45
	Mouse	17.48	312.15
	Monkey	18.28	110.92
CS7171	Human	4.27	407.54
	Mouse	11.42	477.87
	Monkey	8.85	229.15
JKF-034	Human	18.12	95.92
	Mouse	1.16	4,702.82
	Monkey	5.05	401.14

## Discussion

In this study, we tested whether the inhibition of Rac1-dependent active forgetting could ameliorate memory loss in Aβ-based mouse and fly models. We found that Rac1 activity is increased in the hippocampal tissues of AD patients (Fig. 1A) and APP/PS1 mice (Fig. 1D), as well as in whole brain tissues of Aβ42-expressing flies (Fig. S1F), indicating that Rac1-dependent forgetting was enhanced. Such elevations may result from Aβ-dependent stimulation since Aβ42 is capable of activating Rac1 in a cultured cell line (Fig. 1F). This increase is rather specific to Rac1 because the activity levels of RhoA and Cdc42, the other two members of the RhoGTPase family, were not altered in hippocampal tissues of APP/PS1 mice (Fig. S1A–E). This elevated Rac1 activity is likely to accelerate the decay of hippocampus-dependent spatial memory in mice and aversive olfactory memory in flies, since the inhibition of Rac1 activity genetically and pharmacologically rescued memory loss (Figs. 2B–F and 4B). The Rac1 effects were also confirmed at the cellular level for the phenotype of faster decay in LTP observed in mouse brain slices, which was also rescued by both genetic and pharmacological inhibition of Rac1 activity (Fig. 5). Moreover, two small molecular compounds identified through behavioral screening, which were also reported in a previous study (Wang et al., 2012), not only rescued spatial memory, but were also capable of inhibiting Rac1 activity (Fig. 6). Taken together, our data strongly demonstrate that enhanced Rac1 activity contributes to AD-related memory loss and that the pharmacological inhibition of Rac1 activity may be a potentially effective therapeutic approach.

To minimize variations across the behavioral and biochemical assays, we maintained all fly genotypes in an isogenic background through five generations of backcrosses (see Materials and Methods). Appropriate controls were included, such as mice which received vehicle solution and underwent sham surgery, in the respective assays. In all biochemical experiments on Rac1 activity, we included assays of total Rac1 and actin expression levels. This was done so that the level of Rac1-GTP, determined using Western blot, could be used to assess Rac1 activity when the ratio of total Rac1 to actin is not significantly different among the groups (Fig. 1B). Doing this allowed us to obtain highly consistent results from different animal models and from using different approaches, including biochemical, electrophysiological, and behavioral tests.

### Rac1 activation in AD and associated memory loss

A number of previous studies reported that Rac1 expression level, Rac1 activity, and activities of its downstream components, such as PAK and cofilin, are reduced in brain tissues obtained from either AD patients (Zhao et al., 2006; Huang et al., 2013; Kim et al., 2013; Borin et al., 2018) or from APP(swe) Tg2576 mice (Petratos et al., 2008), which is contradictory to our findings. Such discrepancies are likely to arise from a number of sources. First, all studies which reported reduced activity of either Rac1, PAK or cofilin involved either human cortical tissues (Zhao et al., 2006; Huang et al., 2013; Kim et al., 2013; Borin et al., 2018) or multiple brain regions in APP(swe) Tg2576 mice, including the entire cortex (Petratos et al., 2008). However, biochemical assays in this study were confined to hippocampal tissues. In fact, we also assayed PAK activity in the human prefrontal cortex, and Rac1, PAK and cofilin activities are indeed significantly reduced in cortical tissues (Fig. S1G). Second, in the present study, the hippocampus is the brain region which is more important for recent memory formation (Kitamura et al., 2017) and therefore more relevant to early symptoms of memory loss in AD patients (Mufson et al., 2015). Recent spatial memory and daily episodic memory are thought to be formed and stored first in the hippocampus and only remote memory is transferred to the prefrontal cortex (Kitamura et al., 2017). Third, the function of Rac1 activity is distinct in different brain regions. For instance, while Rac1 is involved in forgetting, but not memory formation, in the hippocampus (Liu et al., 2016), it participates critically in acute memory formation in the amygdala (Gao et al., 2015). For these reasons, it was necessary to restrict biochemical assays and Rac1 manipulations to the hippocampus in the present study.

Rac1 is known to activate a number of downstream pathways. It activates the Rac/WAVE complex-Arp2/3 complex pathway to promote actin branching (Rouiller et al., 2008; Firat-Karalar and Welch, 2011) and the Rac/PAK/LIMK/cofilin pathway to regulate cytoskeleton dynamics (Bamburg, 1999; Edwards et al., 1999; Pollard et al., 2003). We did not characterize the downstream pathways because we focused on testing whether enhanced Rac1-dependent active forgetting contributes to AD-related “memory loss” and whether the inhibition of Rac1 activity may be an effective treatment for memory-associated symptoms. This study provides multiple lines of evidence supporting the aforementioned hypotheses. In particular, studies which identified effective small molecule compounds through behavioral screening suggest that pharmacological treatments that could attenuate Rac1 activity in the hippocampus would improve the symptoms of memory loss. Rac1 also is an essential component of the NADPH (nicotinamide adenine dinucleotide phosphate) complex/ROS (reactive oxygen species) generation which has been reported to play a role in chronic neurodegenerative disorders (Ma et al., 2017). This finding raises a possibility that ROS-induced modifications may play an important role in Aβ-induced Rac1-dependent forgetting and deficits hippocampal synaptic plasticity.

### Characterization of effective compounds

In the reported behavioral screening (Wang et al., 2012), 2,392 compounds were bought from Timtec compound library without target-presumption and tested in the Aβ42-expressing transgenic fly using aversive olfactory conditioning assay. Seventy-seven compounds showed positive results for rescuing the memory loss. Among the 26 out of 77 compounds tested in APP/PS1 transgenic mice, 13 compounds were effective in rescuing spatial memory defects. In the current work, three compounds were identified; two (CS7171 and JKF-034) were effective in APP/PS1 mice while one (CS7170) was not (Fig. 6B and 6D). Thus, the two effective compounds in APP/PS1 mice inhibited Rac1 activity in hippocampal tissues (Fig. 6A and 6C), while the ineffective compound was unable to inhibit Rac1 activity. In addition, these two compounds did not affect either RhoA or Cdc42 activity (Fig. S4D–F). Moreover, the administration of JKF-034 led to a reduction in Aβ42 accumulation (Fig. S4A–C).

Among these two effective compounds, JKF-034 appears to be more suitable for further drug development because of its high permeability coefficients during the BBB assay and its long metabolic half-life (T_1/2_) (Tables 2 and 4). The previously reported EHop-016 interacts with Vav, a Rac guanine nucleotide exchange factor (Montalvo-Ortiz et al., 2012), whereas the targets of the two newly identified compounds have not yet been determined. Further pharmacological experiments are needed to determine whether these two compounds are druggable.

## Materials and Methods

### Human subjects

All procedures for the collection and assessment of brain tissues were approved by the Institutional Review Board of the Institute of Basic Medical Sciences, Chinese Academy of Medical Sciences (CAMS) and Peking Union Medical College (PUMC), Beijing, China (Approval Number: 009-2014). The tissues were provided by the Human Brain Bank, CAMS & PUMC, Beijing, China. This study was supported by the China Human Brain Bank Consortium; Neuroscience Center, CAMS, and the CAMS Innovation Fund for Medical Sciences (CIFMS). Ante-mortem written informed consent was obtained from both the potential donors and their next-of-kin to ensure that the donation was completely voluntary, and the ethically approved use of brain tissues in scientific research is permitted. The brain tissue autopsy (based on whole body donation) cohort of CAMS/PUMC was collected from 2012 to 2016, in accordance with international standard human brain banking procedure.

### Animals

Double-transgenic AD mice (APPswe/PS1dE9 (APP/PS1), stock No. 004462) and wild-type (WT) mice (C57BL/6J, stock No. 000664) were purchased from the Jackson Laboratory. Mice were housed individually with *ad libitum* access to water and food. They were maintained on a 12-h/12-h light/dark cycle in a controlled environment (temperature, 22–23 °C; humidity, 50%–60%). The Animal Care and Use Committee of JoeKai Biotech LLC approved all animal experiments.

### Drosophila stock

Human Aβ42 transgenic flies (*elav*/*Y*; *UAS-Aβ42*/*+*) and wide-type flies (*elav*/*Y*;*+*/*+*) were maintained as laboratory stocks. Flies were raised and maintained in a controlled environment (temperature, 22–24 °C; humidity, 60%). Pan-neuronal expression of human Aβ42 transgenic flies has been previously described (Iijima et al., 2004; Wang et al., 2012). All flies used for Pavlovian olfactory conditioning were outcrossed for five generations with the isogenic line w^*1118*^(*isoCJ1*).

### Drug treatment of the animals

Flies were treated with EHop-016 (Yong-Can Biotech). The EHop-016 was dissolved in dimethyl sulfoxide (DMSO; Sigma, D8418-100ML) to make a 10-mmol/L stock solution. This solution was stored at −20 °C for later use. Before administration, 4% (*m*/*v*) sucrose solution was used to dilute the stock solution to a concentration of 100 µmol/L (working solution). After the flies were starved for 3 h in empty vials, they were fed with the working solution for 5 h. Feeding was carried out once per day until testing.

Mice were treated with EHop-016 (10 mg/kg), CS7170 (50 mg/kg), CS7171 (50 mg/kg), and JKF-034 (40 mg/kg). These drugs were dissolved in saline (0.9% NaCl) containing 0.5% Tween-80 (Sigma P4780). Each mouse received 200 μL of the solution by intragastric administration once per day (administered at 9:00 am). The drug administration was started seven days before the beginning of the Morris water maze (MWM) task and was continued until the end of the assay. All control groups were fed with vehicle solution.

### Western blot analysis

The hippocampal tissues from human subjects and mice were harvested and stored in a liquid nitrogen tank for biochemical assay. The tissues were homogenized in cold lysis buffer (Merck-Millipore, 20-168). The insoluble debris were removed after centrifugation at 13,000 rpm (for 10 min, at 4 °C). For GTP-bound Rac/Cdc42, lysates were incubated with GST-tagged PAK (p21-activated kinase)-PBD agarose beads (Cytoskeleton, PAK02) overnight at 4 °C. On the second day, the beads were washed three times with lysis buffer before subjecting them to SDS-PAGE (15%) and transferring them onto nitrocellulose membranes (Millipore, HATF00010). The membranes were incubated with primary antibodies (for Rac1: BD Transduction Laboratories, #610650, 1:2,000 dilution; for Cdc42, Santa Cruz Biotechnology, lot No. sc-8401, 1:500 dilution) overnight at 4 °C and with HRP-conjugated goat anti-mouse IgG (Cell Signaling Technology, #7076) for 1 h at room temperature. Intensities of the detected bands were analyzed using ImageJ software (National Institutes of Health). The total amount of Rac1 in the lysate was detected using a routine Western blot procedure. RhoA-GTP levels were measured by using the Rho-GTP monoclonal antibody (New East, lot No. 26904, Dilution: 1:1,000). Information regarding the other antibodies used is as follows: anti-RhoA (67B9), Cell Signaling, lot No. #2117, dilution: 1:1,000; anti-Actin, Clone C4, Millipore, lot No. #3018859, dilution: 1:2,000; anti-Amyloid 1-42 6E10, BioLegend, lot No. 803001, dilution: 1:1,000; anti-Phospho-PAK1 (Ser144)/PAK2 (Ser141), Cell Signaling, lot No. #2606, dilution: 1:1,000.

### Morris water maze (MWM)

The MWM test, a classic paradigm used to study spatial reference memory, was performed (Vorhees and Williams, 2006). Briefly, a circular polypropylene water tank (120 cm in diameter) was filled with opaque water and milk (20 °C ± 1 °C) and a transparent platform (15 cm diameter) was submerged (2 cm below the water surface) in the center of one of the four virtually divided quadrants. Distal cues were fixed on the wall of the tank as spatial references. For the classic training paradigm (Figs. 2 and 6), mice were allowed to find the platform within 60 s and to remain on it for at least 5 s. There were four sessions with 1-h intervals on each training day (paradigm shown in Fig. 2A upper panel). For the modified training paradigm (Fig. 3A), mice were trained over 5 consecutive sessions with a 1-h intervals within one day. After training, mice were returned to their home cages and the memory of the location of the platform was tested at 6 h after training (Fig. 3C–D). A mouse behavior tracking software (ANY-maze software) was used to record data automatically.

### Probe test

At 24 h after the last training, the platform was removed from the tank. Mice in all groups swam freely in the tank for 60 s. The amount of time each mouse stayed in each quadrant was recorded.

### Pavlovian olfactory aversive classic conditioning

To assess learning and memory, flies underwent classic olfactory aversive conditioning. One- to two-day-old flies were collected and fed with EHop-016 (100 µmol/L) for 7 days. Training and testing were performed at 24 °C with 60% relative humidity. During training, about 100 flies were exposed to an electric shock (unconditioned stimulus [US]) paired with either 3-octanol (>95% purity; Fluka, Sigma-Aldrich) or 4-methylcyclohexanol (>99% purity; Fluka, Sigma-Aldrich) as the conditioned stimulus (CS). Performance was tested using a T-maze apparatus within which 3-octanol and 4-methylcyclohexanol were delivered from opposite arms simultaneously. Flies were allowed to choose an arm for 120 s. The learning score was calculated as the difference between the number of flies in each arm divided by the sum of flies in both arms immediately after training. Performance index was calculated as the average of two reciprocal tests to avoid odor bias. For the memory test, trained flies were placed into vials with food. They remained in these vials for 15 min, 30 min, 60 min or 180 min for later testing.

### *In vitro* slice preparation and electrophysiology

Acute 300-μm transverse hippocampal slices were prepared using a vibratome (VTS3000, USA). The slices were maintained at room temperature in a submersion chamber in artificial cerebrospinal fluid (ACSF) containing 125 mmol/L NaCl (Sigma, 71376), 2.5 mmol/L KCl (Sigma, P9541), 2 mmol/L CaCl_2_ (Sigma, 499609), 1 mmol/L MgCl_2_ (Sigma, M1028), 1.25 mmol/L NaH_2_PO_4_ (Sigma, S3139), 24 mmol/L NaHCO_3_ (Sigma, S5761) (pH = 7.4), and 15 mmol/L glucose (Sigma, G8270), bubbled with 95% O_2_ and 5% CO_2_. Slices were incubated for at least 1 h before removal for experiments. Slices were kept in the MED probe for 1 h for attachment to the 16-channel array (MED-PG515A, Alpha MED Sciences; preheated to 33 °C). Extracellular field excitatory post-synaptic potentials (fEPSPs) in the Schaffer collateral pathway were evoked at 0.025 Hz and recorded in the CA1. The fEPSPs were evoked using a stimulation intensity that elicited a 30% maximal response. Data acquisition and analysis were performed using the multi-electrode MED64 hardware and software package (Panasonic). Additional analyses were performed using custom macros running under Igor Pro. The LTP was induced by one train of theta burst stimulation (TBS) consisting of one epoch of 10 trains of five bursts repeated at 5 Hz and delivered at the same intensity as in the baseline recordings. Data collection and analysis were performed by experimenters who were blind to the conditions of the experiments.

### DNA constructs and recombinant adeno-associated virus (rAAV) production

All plasmids (CaMKIIα-Rac1 dominant negative-green fluorescent protein (GFP); CaMKIIα-GFP) were constructed according to standard molecular biology procedures and subsequently verified by double-strand DNA sequencing. The embryonic kidney cell line (HEK-293) was co-infected with adenoviral helper plasmids for AAV production. The virus was diluted and titer-matched to 5.0 × 10^12^ genome copy (GC) per mL using phosphate-buffered saline (PBS) before injection.

### Stereotactic surgery procedure

Pentobarbital (Sigma) 0.2% (*m*/*v*) with PBS as solvent (pH = 7.4) was peritoneally injected into mice (200 μL per mouse) 5 min before surgery. Bilateral craniotomies were performed using a 0.5-mm diameter drill. Subsequently, 400 nL and 600 nL of virus were injected into the DG (−2.2 mm AP, +/− 1.5 mm ML, −2.0 mm DV) and CA1 (−2.2 mm AP, +/− 1.5 mm ML, −1.5 mm DV), respectively. After surgery, mice were returned to their home cages. They were allowed a two-week recovery period before behavioral experiments were performed. A representative injection location is shown in Fig. S2A. All control groups underwent sham surgeries.

### Cell assay

Synthetic wide-type Aβ42 (purchased from AnaSpec) was initially dissolved in hexafluoroisopropanol (Sigma) to 5 mg/mL for subpackaging. Hexafluoroisopropanol was removed by air drying to obtain an Aβ42 peptide film, which was stored at −80 °C. Then, 24 h before the experiment, the peptide film was first re-suspended in DMSO to a concentration of 12.5 mg/mL and then diluted with Dulbecco’s Modified Eagle Medium (DMEM)/F-12 (phenol red-free; Invitrogen) to a final concentration of 500 μg/mL. The HEK-293 cells were cultured in DMEM containing 10% fetal bovine serum (FBS) at 37 °C with 5% CO_2_. For oligomer treatment, cells were washed with fresh medium and incubated with 10 μg/mL Aβ42 oligomers for 2 h. Cells were washed twice with PBS before they are collected for Rac1 activity detection by G-LISA (BK128, Cytoskeleton) in accordance with the manufacturer’s protocol.

### *In vitro* blood-brain barrier permeability assay

The MDR-MDCK cell line can be used as a fast blood-brain barrier (BBB) model to aid drug discovery (Wang et al., 2005). In this study, the MDR-MDCK cell line was used to evaluate the permeability coefficients of CS7171 and JKF-034. Briefly, CS7171 and JKF-034 were prepared to final concentrations of 5 μmol/L. The cells were dosed on the basolateral side (B-A) and apical side (A-B) and incubated at 37 °C for 90 min. Each test was performed in duplicate. The apparent permeability coefficient, P_app_, was calculated using the following formula:

$${\text{P}}_{\text{app}} = \, \left( {{\text{V}}_{\text{A}} /\left( {{\text{Area }} \times {\text{ Time}}} \right)} \right) \, \times \, (\left[ {\text{drug}} \right]_{{{\text{acceptor}},{ 9}0 - { \hbox{min} }}} /\left( {\left( {\left[ {\text{drug}} \right]_{{{\text{donor}}, \, 0 - { \hbox{min} }}} } \right) \, \times {\text{ Dilution Factor}}} \right)$$

V_A_ is the volume in the acceptor well, Area is the surface area of the membrane, Time is the total transport time in seconds, [drug]_donor, 0-min_ is the donor drug concentration measured at 0 min; [drug]_acceptor, 90-min_ is the acceptor drug concentration measured at 90 min; dilution factor is the degree of dilution of the donor sample before loading to LC-MS/MS. If the measured permeability coefficient (P_app_) is greater than 10 × 10^−6^ cm/s, it is highly likely that this compound could be efficient in crossing the BBB. Conversely, compounds with P_app_ values lower than 5 × 10^−6^ cm/s may have low permeability. A P_app_ value of 5–10 × 10^−6^ cm/s indicates moderate permeability. Metoprolol was used as a highly permeable control, while atenolol was used as a low-permeability control.

### Pharmacokinetics study

For the CS7171 and JKF-034 pharmacokinetics (PK) assays, 30 CD1 mice were purchased from JH Laboratory Animal Co. LTD (Qualification No: SCXK (SH) 2017-001220170012002565). A total of 18 CD1 mice were grouped for intravenous injection (I.V) with 10 mg/kg CS7171 and 2.5 mg/kg JKF-034. Sampling was performed at 0.083, 0.25, 0.5, 1, 3, 6 and 24 h after administration. A total of 12 CD1 mice were grouped for oral administration (P.O) of 50 mg/kg CS7171 and 40 mg/kg JKF-034. Sampling was performed at 0.25, 0.5, 1, 3, 6 and 24 h after administration. The blood samples were maintained in wet ice first and centrifuged to obtain plasma within 15 min post-sampling. Subsequently, the samples were subjected to LC-MS/MS analysis.

### In vitro metabolism stability in liver microsomes

Measurements of metabolic stability can be performed with liver microsomes, which are known to contain the major drug-metabolizing enzymes and UDP-glucuronosyltransferase (Baranczewski et al., 2006). The *in vitro* metabolic stability levels of CS7171 and JKF-034 were measured using human, monkey, and mouse liver microsomes. The metabolic half-time (T_1/2_) and intrinsic clearance (Cl_int_) were assayed using LC-MS analysis. Ketanserin is a potent, orally effective antagonist of endogenous serotonin. Its pharmacological profile is well studied (Brogden and Sorkin, 1990). In this study, we used ketanserin as a control. If the T_1/2_ of the tested compound was 30–120 min, the compound was considered to show moderate metabolism in liver microsomes. If the T_1/2_ value was less than 30 min, the compound was considered to be susceptible to metabolism in liver microsomes.

### Statistical analysis

All data were analyzed using a nonparametric *t*-test, or post hoc test following anslysis of variance (ANOVA). All analyses were performed using GraphPad Prism 6. Statistical results are presented as the mean ± standard error of the mean (SEM). A *P* < 0.05 was considered statistically significant. An asterisk was used to indicate statistically significant *P* values.

## Electronic supplementary material

Below is the link to the electronic supplementary material.

Supplementary material 1 (PDF 476 kb)
