# Supplementary material for: Inhibition of Rac1-dependent forgetting alleviates memory deficits in animal models of Alzheimer’s disease
Source: Protein Cell. 2019 Jul 18;10(10):745–59. doi: 10.1007/s13238-019-0641-0 (PMC6776562; doi:10.1007/s13238-019-0641-0)
Supplement: Supplementary file 1 — Supplementary material 1 (PDF 476 kb) [file 13238_2019_641_MOESM1_ESM.pdf]

Supplementary Materials

Supplementary Figure 1. The activity of other GTPases in the hippocampus of APP/PS1 mice and the elevation of Rac1 activity in the whole brain of AD flies

A Representative Western blot results for RhoA-GTP, Cdc42-GTP, RhoA, and Cdc42 in the hippocampus of 8-month-old APP/PS1 mice and age-matched WT mice.

B No difference in RhoA activity was observed in the hippocampus between APP/PS1 and WT mice (n.s, $P > 0.05$, *t*-test, two-tailed; n = 4 per group).

C No difference in the amount of RhoA was observed in the hippocampus between APP/PS1 and WT mice (n.s, $P > 0.05$, *t*-test, two-tailed; n = 4 per group, same mice as B).

D No difference in Cdc42 activity was observed in the hippocampus between APP/PS1 and WT mice (n.s, $P > 0.05$; *t*-test, two-tailed; n = 4 per group, same mice as B).

E No difference in the amount of Cdc42 was observed in the hippocampus between APP/PS1 and WT mice (n.s, $P > 0.05$; *t*-test, two-tailed; n = 4 per group, same mice as B).

F Exacerbated activation of Rac1 activity in the elder Tg-AD fly model (**$P < 0.01$, *t*-test, two-tailed; n = 6 for *elav/Y; +*; n = 10 for *elav/Y; UAS-Aβ42/+*).

G The activity of PAK was significantly decreased in the human prefrontal cortex. Representative Western blot of phosphorylated PAK and total PAK (*$P < 0.05$, *t*-test, two-tailed; n = 4 for each group).

Data information: All values are expressed as mean ± SEM.

Supplementary Figure 2. The location of injection in the hippocampus and the escape latency during the MWM

A To verify that the hippocampus is the location where the rAAV was injected and that Rac1 DN is expressed in excitatory neurons. Representative confocal data show that the green fluorescent signal under the control of CaMKII α was strictly expressed in the hippocampus , mainly in the dentate gyrus (DG), scale bar: 1 mm.

B Two-hour memory and four-hour memory was retained in 3-month-old APP/PS1 mice after 6 training sessions in one day ($P > 0.05$, two-way ANOVA followed by Tukey's multiple comparison test; n = 8 per group)

Data information: All values are expressed as mean ± SEM.

Supplementary Figure 3. Structure of CS7171, CS7170, and JKF-034

A Chemical structure of CS7171.

B Chemical structure of CS7170, which is an analogue of CS7171.

C Chemical structure of JKF-034.

Supplementary Figure 4. Effects of CS7171 and JKF-034 on Aβ42 aggregation and clearance and on other RhoGTPase activities

A Representative Western blot of Aβ42 detected by Aβ42 monoclonal antibody (6E10) after two weeks of administration (P.O) of CS7171 and JKF-034 in 7-month-old APP/PS1 mice.

B The level of Aβ42 monomer was decreased by JKF-034. CS7171 did not affect the level of Aβ42 monomer (*$P < 0.05$; n.s, $P > 0.05$, one-way ANOVA followed by Tukey's multiple comparison test; n = 4 for APP/PS1, n = 6 for CS7171, n = 5 for JKF-034).

C The levels of the oligomer form of Aβ42 were not changed by CS7171 and JKF-034 (n.s, $P > 0.05$, one-way ANOVA followed by Tukey's multiple comparison test; n = 4 for APP/PS1, and n = 6 for CS7171 and JKF-034, same mice as B).

D Representative Western blot of total RhoA, total Cdc42, RhoA-GTP, and Cdc42-GTP after two weeks of drug administration (P.O) of CS7171 and JKF-034 in 7-month-old APP/PS1 mice.

E Cdc42 activity was not changed by the administration of CS7171 and JKF-034 (n.s, $P > 0.05$, one-way ANOVA followed by Tukey's multiple comparison test; n = 3 for each group).

F RhoA activity was not altered by the administration of CS7171 and JKF-034 (n.s, $P > 0.05$, one-way ANOVA followed by Tukey's multiple comparison test; n = 3 for each group, same mice as E).

Data information: All values are expressed as mean ± SEM.

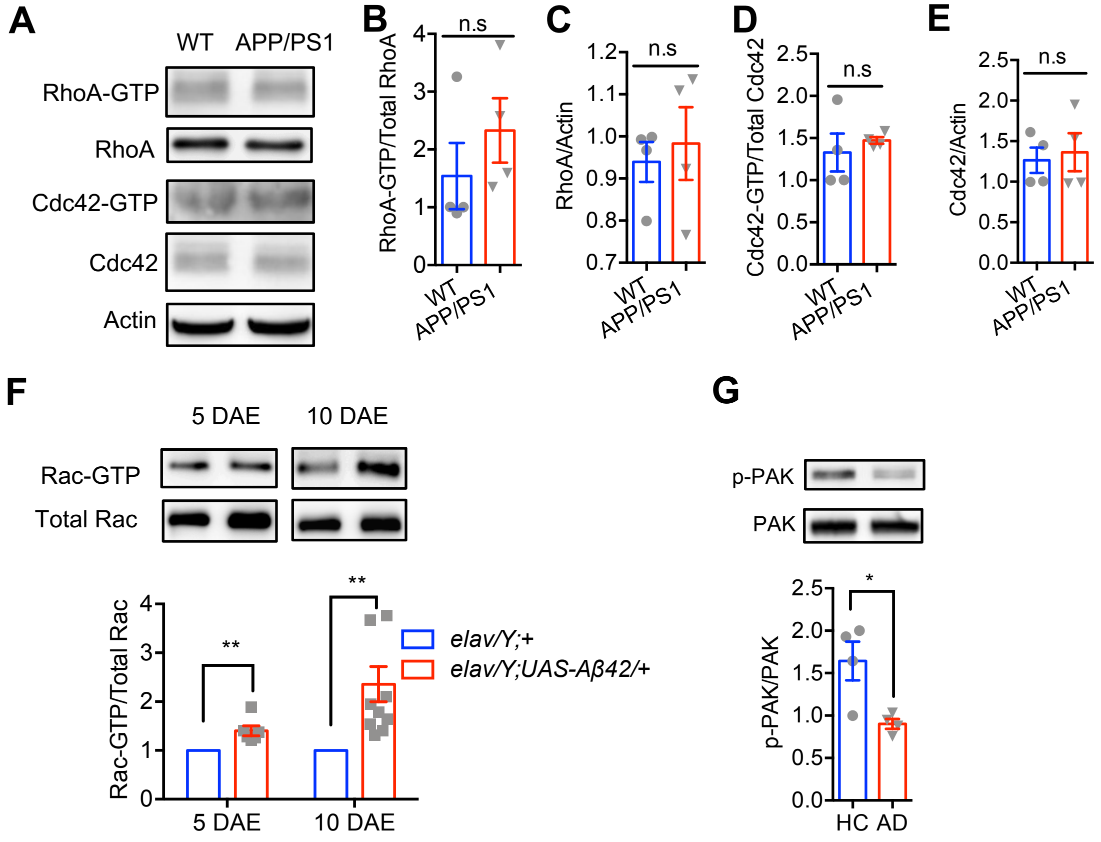
A
WT
APP/PS1
RhoA-GTP
RhoA
Cdc42-GTP
Cdc42
Actin
B
n.s
RhoA-GTP/Total RhoA
WT
APP/PS1
C
n.s
RhoA/Actin
D
n.s
Cdc42-GTP/Total Cdc42
E
n.s
Cdc42/Actin
F
5 DAE
10 DAE
Rac-GTP
Total Rac
**
**
elav/Y;+
elav/Y;UAS-Aβ42/+
Rac-GTP/Total Rac
G
p-PAK
PAK
*
p-PAK/PAK
HC
AD

Figure S1

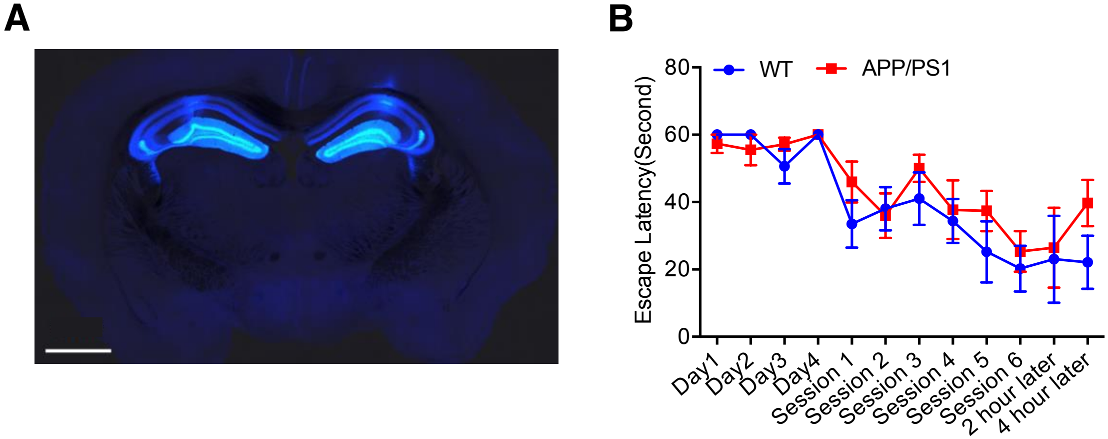
A
B
WT
APP/PS1
Escape Latency(Second)
80
60
40
20
0
Day1
Day2
Day3
Day4
Session 1
Session 2
Session 3
Session 4
Session 5
Session 6
2 hour later
4 hour later

Figure S2

A

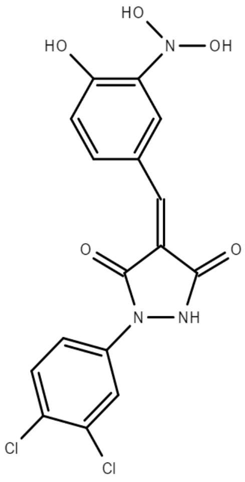

CS7171

B

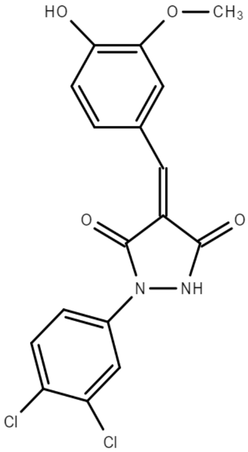

CS7170

C

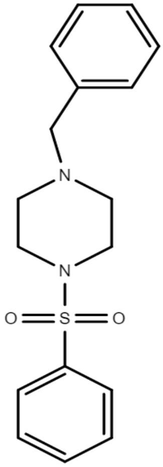

JKF-034

Figure S3

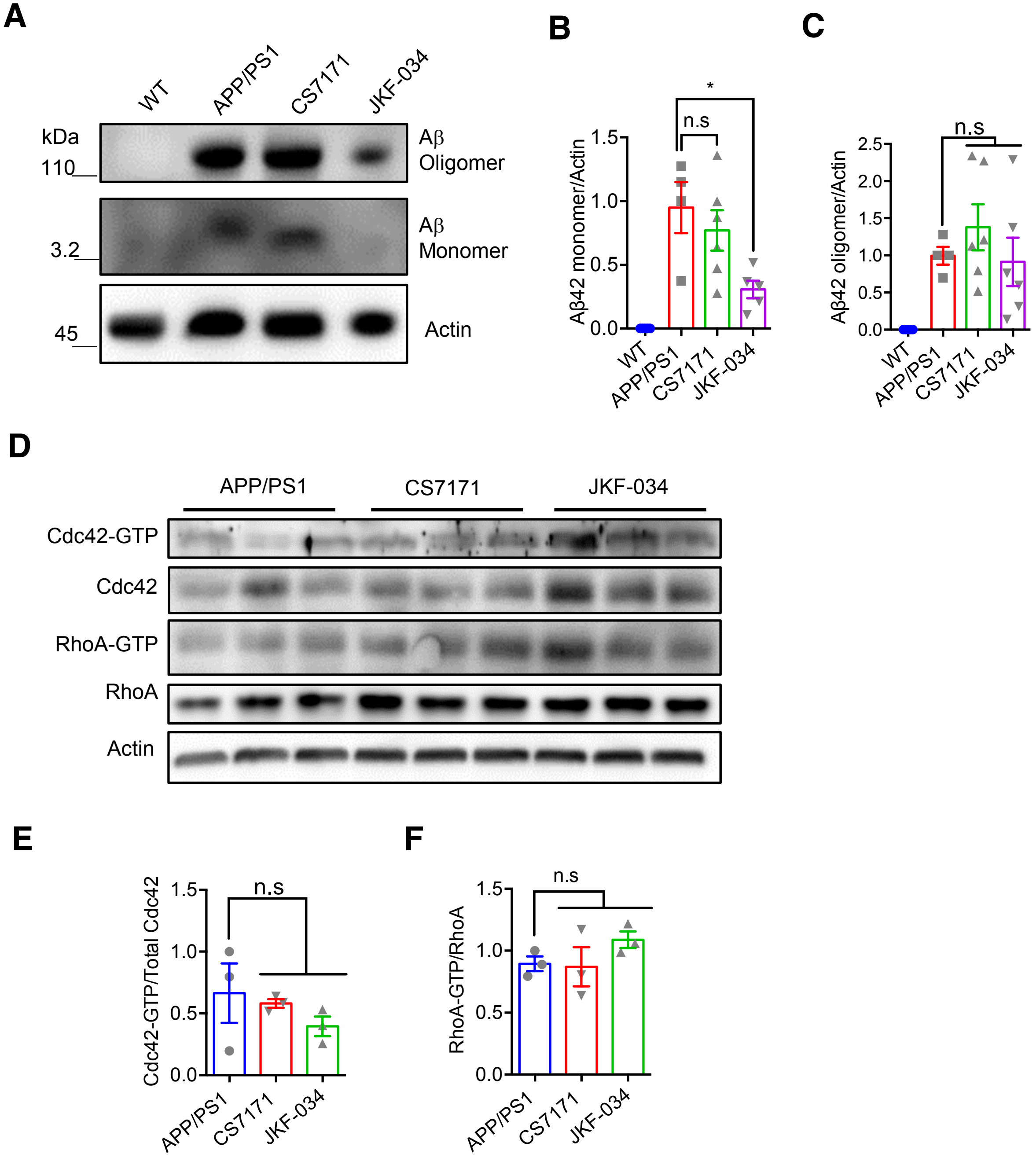
A
WT
APP/PS1
CS7171
JKF-034
kDa
110
3.2
45
Aβ Oligomer
Aβ Monomer
Actin
B
Aβ42 monomer/Actin
n.s
*
C
Aβ42 oligomer/Actin
n.s
D
APP/PS1
CS7171
JKF-034
Cdc42-GTP
Cdc42
RhoA-GTP
RhoA
Actin
E
Cdc42-GTP/Total Cdc42
n.s
F
RhoA-GTP/RhoA
n.s

Figure S4
